# A 2-year follow-up of the effects of combined binge drinking and cannabis consumption on academic performance and adjustment in Spanish third-year university students

**DOI:** 10.3389/fpsyg.2023.1223597

**Published:** 2023-08-03

**Authors:** María Fernanda Páramo, Fernando Cadaveira, María Soledad Rodríguez

**Affiliations:** ^1^Department of Developmental and Educational Psychology, Faculty of Psychology, Santiago de Compostela, Spain; ^2^Department of Clinical Psychology and Psychobiology, Faculty of Psychology, Santiago de Compostela, Spain; ^3^Department of Social, Basic Psychology and Methodology, Faculty of Psychology, Santiago de Compostela, Spain

**Keywords:** follow-up, co-consumption, academic performance, adjustment, university students

## Abstract

**Introduction:**

The study was based on 2-year follow-up of the effects of binge drinking and cannabis co-consumption on academic performance and adjustment in Spanish Third-Year University Students and to further explore the impact of academic adjustment on this relationship.

**Methods:**

A total of 144 students (aged 19–20 years) enrolled in the third year of university completed the study. The students were recruited during in first academic year (T1) via a survey that included items regarding the use of alcohol (AUDIT-C), cannabis and other drugs and demographic variables. Then, participants meeting the study criteria were then selected and invited by e-mail to a clinical (face-to face) structured interview. The participants completed a calendar of alcohol consumption during the 6 months prior to the interview (Alcohol Timeline Follow back), and recorded cannabis consumption in 3 months prior to the interview. To examine the effects of alcohol and cannabis co-consumption on the outcome variables, we categorized participants into three consumption groups (i.e., control, BD, and BDCA) based on the number of BD days and cannabis unit scores.

**Results:**

Binge drinking and cannabis co-consumption in first-year students was significantly associated with poor academic performance and adjustment after 2 years of undergraduate study. Relative to controls, co-consumers (BDCA) reported significantly lower academic and personal-emotional adjustment to university as well as poorer performance. Mediation analysis showed that academic adjustment explains the mechanism by which BDCAs perform less well, mediating the relationship between co-consumption and academic performance, with an indirect effect representing 64.61% of the total effect. Furthermore, the mediating effect of academic adjustment was maintained after controlling for academic adjustment and baseline grade point average (T1).

**Conclusion:**

This prospective follow-up study helps to further our knowledge of how combined binge drinking and cannabis consumption may affect university adjustment and academic success in Spanish university students Overall, the study results should encourage health professionals, educational psychologists and academic institutions to take ownership of the need for support and involvement in prevention, as well as for provision of guidelines for implementing appropriate intervention strategies.

## 1. Introduction

Studies on the short- and long-term effects of combined binge drinking (BD) and cannabis consumption on academic performance in university students are underrepresented (Arria et al., 2013; Meda et al., 2017) even though academic performance is known to act as a barometer for educational success or ineffectiveness and as a key predictor of the prospects of young adults (Jalaliyoon and Taherdoost, 2012). The lack of relevant studies is especially surprising if we consider that the co-use of legal and illegal drugs in the university population has increased substantially in Western countries in the last decade (Substance Abuse and Mental Health Services Administration, 2020).

The sociocultural, economic, and demographic context of our time, together with the academic standards prevailing in Western universities, has in recent decades generated a system that requires students to assume greater autonomy in decision making and a higher level of competitiveness that today largely depends on their ability to work with information (Staff et al., 2010). University students find themselves at the *emerging adulthood* stage (Arnett and Jensen, 2000). This is an intermediate developmental stage, situated between adolescence and adulthood proper, characterized by identity discovery, uncertainty about sexual beliefs, evaluation of different options and decisions about priorities in a gradual and responsible way. It is also a period in which the students must balance their responsibilities to their parents with their desire for freedom from social roles and normative expectations in an unknown and highly competitive environment (Arnett, 2006; Arnett et al., 2011; Peer and McAuslan, 2016; Scales et al., 2016). In this context of uncertainty and identity confusion together with permissive policies (Vitale, 2014; Bolin et al., 2017; De Faria et al., 2021), peer pressure, and low risk perception (Schulenberg et al., 2017), some risk behaviors such as substance use, are clearly increasing (Johnston et al., 2009; Skidmore et al., 2016; Allen et al., 2017; Gómez et al., 2017; Holloway and Bennett, 2018; Welsh et al., 2019; Jackson et al., 2020).

Access to university is no longer a protective factor for many students (Schulenberg and Maggs, 2002; Sarokhani et al., 2013; Eskin et al., 2016; Ribeiro et al., 2018; Arria et al., 2020; Thompson et al., 2021). Although the transition to university is not a homogeneous experience (Mayhew et al., 2005; Fierro Arias and Moreno Hernández, 2007; Coertjens et al., 2017), students generally face constant demands of academic, personal, emotional, and social adjustment for which they are not always prepared (Baker and Siryk, 1989; Harvey et al., 2006; Credé and Niehorster, 2012). University adjustment is more than a score on a standardized test, and it involves facing multiple stressors that make the initial entry period particularly difficult time, which can lead to high levels of anxiety, interpersonal conflicts, feelings of isolation and loneliness, constant feelings of being overwhelmed, poor adaptation, academic failure and attrition (Abdullah et al., 2009; Aspelmeier et al., 2012; Clinciu, 2013; Bailey and Phillips, 2016; Rodríguez et al., 2017; Sharp and Theiler, 2018; Pascoe et al., 2020; Raza et al., 2021). Students face a myriad of challenges during their transition to university, including developmental changes, which make them particularly vulnerable to substance abuse during this period (Staff et al., 2010; Derefinko et al., 2016). BD and cannabis consumption some of the most influential risk factors (Wechsler et al., 2002; Caldeira et al., 2008; Arria et al., 2015; Subbaraman and Kerr, 2015; Hayaki et al., 2016; Merrill and Carey, 2016; O’Hara et al., 2016; Gómez et al., 2017; Johnson et al., 2017; Meda et al., 2017; Busto Miramontes et al., 2019; Mallett et al., 2019). The national survey on drugs in Spain (Substance Abuse and Mental Health Services Administration, 2020) reports frequency of BD in the population aged between 14 and 18 years of 32.3%, that almost half of this population are cannabis users (OEDA, 2019).

Aside from impairing overall health (Simons et al., 2005; Hall and Degenhardt, 2009; Keith et al., 2015) and well-being (Liu et al., 2019), prior cross-sectional (Aertgeerts and Buntinx, 2002; Wolaver, 2002; El Ansari et al., 2013; Liguori and Lonbaken, 2015; Piazza-Gardner et al., 2016; Bolin et al., 2017; Tembo et al., 2017; Hernández-Serrano et al., 2018; Páramo et al., 2020) and longitudinal (Arria et al., 2013, 2015; Suerken et al., 2016; Meda et al., 2017; Patte et al., 2017) research has found that students who partake in binge drinking and cannabis use, either together or separately, spend less time studying, miss more classes, are less academically motivated, obtain lower grade point averages (GPAs), and also have an elevated risk of university attrition. Previous data obtained by our research group (Páramo et al., 2020) in a cohort of Spanish first-year university students classified as binge drinking and cannabis users have shown that academic adjustment mediates the effects of BD and cannabis on academic performance. The study demonstrated the importance of poor academic adjustment as a risk factor for academic performance.

The impact of both BD and cannabis consumption on academic performance is associated with adverse effects on cognitive functions such as inhibitory control (López-Caneda et al., 2014), impairments in memory, particularly verbal and working memory (Solowij and Battisti, 2008; Deniel et al., 2021; Morie et al., 2021), attentional deficits, and poorer processing speed (Crean et al., 2011; Jacobus et al., 2015; Gil-Hernandez et al., 2017; Carbia et al., 2018).

The effects of combined BD and cannabis consumption on academic performance and adjustment in university students have mainly been examined in cross-sectional studies and it is difficult to observed changes in different cohorts. Most of the few longitudinal studies reported in the above-mentioned literature have been carried out in the USA. To the best of our knowledge, there are no follow-up studies with data obtained at two time points and that analyze the impact of combined BD and cannabis consumption on academic performance and adjustment in Spanish university students. Therefore, the primary goal of the present study was to follow-up the effects of combined BD and cannabis consumption in first-year students on academic performance and adjustment after 2 years of undergraduate study. The second goal was to analyze the mediating role of academic adjustment on the relationship between combined BD /cannabis consumption and GPA. The final goal was to analyze whether, after controlling for T1 academic adjustment and GPA, the effects of co-consumption on academic performance are maintained. Based on previous findings, it was hypothesized that: (1) BD and cannabis co-consumption would be associated with poorer long-term GPA; (2) BD and cannabis co-consumption would be associated with poorer long-term university adjustment; and (3) we expected that the academic adjustment will mediate the relationship between BD and cannabis co-consumption and GPA, once controlled the effect of academic adjustment and baseline grade point average.

## 2. Materials and methods

### 2.1. Participants and procedure

The study was a follow-up with two measurement points: T1, in the students’ first year at university and T2 in the student’s third year at university (approximately 2 years later). Participants were recruited during their first year at university (T1) through a survey including questions about use of alcohol (AUDIT-C) (Saunders et al., 1993; Varela et al., 2005), cannabis and other drugs and also about demographic variables. Participants who met the study criteria were then selected and invited by e-mail to a structured clinical (face-to face) interview.

Students completed a calendar of alcohol consumption during the 6 months prior to the interview (Alcohol Timeline Followback, TLFB) (Sobell and Sobell, 1992), and also provided a record of their cannabis consumption in 3 months prior to the interview. In order to examine the effects of alcohol and cannabis co-consumption on our outcome variables, we categorized participants into three consumption groups (i.e., control, BD, and BDCA) based on the number of days BD and cannabis unit scores. Male students who consumed 6 or more standard drinks and female students who consumed 4 or more standard drinks on a single occasion, at least once in the last 30 days, were classified as binge drinkers. The BD-cannabis (BDCA) group consisted of BD students who also consumed at least 3 cannabis units in the last 3 months.

Exclusion criteria for both groups were as follows: scores above 20 in the AUDIT; scoring in at least 2 symptomatic dimensions of the SCL- 90-R; uncorrected sensory deficits; personal history of traumatic brain injury or neurological disorder, personal history of any neurological or DSM-IV axis I disorder in first-degree relatives, family history of alcoholism in first-degree relatives; consumption of illegal drugs, other than cannabis.

Students assessed at T1 provided a phone number and/or e-mail address for the follow-up study. Only those participants who attended the previous evaluation (and met the inclusion criteria) were contacted again to participate in the follow-up. The individuals received monetary compensation (30 euros) for participation and gave their written informed consent, including consent to obtain their academic records. The research was approved by the University of Santiago de Compostela bioethics committee.

Of the 258 students assessed at T1, 166 students participated at T2, i.e., the participation rate follow-up was 64.34%. Of these students, 7 were excluded because they did not meet the inclusion criteria regarding personal data, and another 15 were excluded either because they did not complete the third year of university degree, or some GPA data were missing.

The final sample included 144 students enrolled in the third year of a degree course at the University of Santiago de Compostela, in the academic year 2018–2019. Participants ranged in age between 19 and 20 years (*M* = 19.92, SD = 0.26); 76 were woman and 68 males. Most of the students were single (98.6%), not working (86.8%) and their families were of medium socioeconomic level (84.3%).

Respondents who participated in their third year (*n* = 144) did not differ from non-respondents (*n* = 114) regarding sociodemographic variables (gender, mother’s and father’s educational level, mother’s and father’s employment, socioeconomic status and residence) and related consumption variables (age of onset of alcohol use, AUDIT-C, number of days BD, average number of drinks per drinking day, and average number of cannabis units) at Time 1. In addition, the proportion (%) of students in each consumption group (C, BD, BDCA) was similar in participants and non-participants.

### 2.2. Measures

In their first year at university (T1), the participating students completed a questionnaire that included questions on sociodemographic variables: age, gender, mother’s and father’s educational level (primary school, high school, university), mother’s and father’s employment (unemployed, employed) and place of residence (in family home, away from family home). It also included questions on the following variables related to substance use: age of onset of alcohol use, AUDIT-C scores, number of days BD, average number of drinks per drinking day, and cannabis units.

The Spanish validated version of the Student Adaptation to College Questionnaire (SACQ) (Baker and Siryk, 1989; Rodríguez González et al., 2012) was used to assess students’ academic, social, personal-emotional, and institutional adjustment. The scale was administered at the end of participants’ first semester, at T1 and T2. This scale consists of 67 items responded on a 9-point scale ranging from 1 = *strongly disagre*e to 9 = *strongly agree*. Negatively worded items were reverse-scored so that higher scores denoted better adjustment and mean scores were used for each following subscale. Academic adjustment (24 items, e.g., “I have been keeping up to date on my academic work”) involves coping with the academic demands of the university experience. Social adjustment (20 items, e.g., “I feel that I fit in well as part of the college environment”) assesses students’ feelings of fitting in, participating in social activities and making friends at university. Personal-emotional adjustment (15 items, e.g., “I have been feeling tense or nervous lately”) focuses on the student’s psychological state and the extent to which he or she is experiencing general psychological distress, and institutional adjustment (15 items, e.g., “I wish I were at another college or university”) assesses attachment to the specific institution the student is attending, and the quality of the relationship between the student and the institution. Cronbach α was 0.90 for T1 and T2 academic adjustment, 0.84 and 0.82 for T1 and T2 social adjustment, 0.85 and 0.87 for T1 and T2 personal–emotional adjustment, and 0.83 and 0.76 for T1 and T2 institutional attachment.

Academic achievement was assessed by the students’ GPAs obtained from the university’s central administration system at the end of first and thirst year. In the Spanish education system, grades are scored from 1 to 10, and a score of 5 or higher is required to pass.

### 2.3. Analyses

Descriptive statistics for baseline (T1) of the participants in each consumer group were first calculated. Chi-square analyses were conducted to examine differences between groups in sociodemographic variables. One-way ANOVA and Scheffe’s *post-hoc* test were used to examine differences in variables related to substance use, adaptation to university and GPA. Second, One-way ANOVA was then used to assess the effect of alcohol and cannabis use evaluated at T1 on dimensions of adjustment to university (academic, social, personal-emotional adjustment and institutional attachment) and GPA measured at T2. Test results were considered statistically significant at 𝑝 ≤ 0.05. Finally, mediation analysis with the PROCESS macro (Hayes, 2017) was used for 10,000 bootstrap samples with a 95% bootstrap confidence interval (CI) to assess whether alcohol and cannabis consumption were associated with poor academic achievement indirectly through its effect on academic adjustment. In the first model, the C vs. BDCA group (T1) served as dichotomous independent variable, the average GPA (T2) served as the dependent variable, and academic adjustment (T2) was the mediator. To improve modeling of the change over time in mediator and outcome variables for predicting later measures in T2, academic adjustment and baseline GPA (T1) were included as covariates in a second model, as suggested by Darlington and Hayes (2017). Data were analyzed using the IBM Statistical Package SPSS Version 28.0.

## 3. Results

### 3.1. Baseline characteristics of the participants

Descriptive statistics of the participants in each consumer group (Control, BD, BDCA) are presented in Table 1. Chi-square tests showed that there were no significant differences in sociodemographic variables. The ANOVA showed differences in means between all groups in variables related to substance use; the BDCA group had the highest mean scores in AUDIT-C, number of BD days, average number of drinks per drinking day, cannabis units and the lowest mean scores in age of onset of alcohol use. In addition, higher consumption of alcohol and cannabis was significantly associated with lower GPA (*F*_2, 143_ = 6.67). *Post-hoc* tests indicated significant differences in mean academic achievement between BDCA and Control groups (−1.02 in a range from 1 to 10).

**Table 1:** Characteristics of the overall sample and each consumer group [% or *M* (SD), and *p*-values for comparative statistics].

Variable	Total (*N* = 144)	Control (*n* = 55)	BD (*n* = 54)	BDCA (*n* = 35)	*p*-value
**Sociodemographic**	%	%	%	%	
**Gender**					
Male	47.2	49.1	50.0	40.0	0.61
Female	52.8	50.9	50.0	60.0	
**Mother’s educational level**					
Primary school	34.8	40.0	33.3	28.6	0.33
High school	24.8	18.2	33.3	22.9	
University	40.4	41.8	33.3	48.6	
**Father’s educational level**					
Primary school	37.0	37.0	42.0	29.4	0.44
High school	28.3	33.3	26.0	23.5	
University	34.8	29.6	32.0	47.1	
**Mother’s employment**					
Unemployed	20.9	25.5	20.4	14.3	0.44
Employed	79.1	74.5	79.6	85.7	
**Father’s employment**					
Unemployed	10.3	13.2	8.2	8.8	0.67
Employed	89.7	86.8	91.8	91.2	
**Socioeconomic status**					
Low	11.4	13.0	13.5	5.9	0.23
Medium	84.3	87.0	80.8	85.3	
High	4.3	0	5.8	8.8	
**Residence**					
In family home	16.0	21.8	16.7	5.7	0.12
Away from home	84.0	78.2	83.3	94.3	
**Substance use**	*M* (SD)	*M* (SD)	*M* (SD)	*M* (SD)	
Age of onset of alcohol use	15.70 (1.24)	16.39 (1.00)	15.57 (1.28)	15.12 (1.09)	<0.001
AUDIT-C^a^	3.89 (2.59)	1.48 (1.57)	4.71 (1.88)	6.37 (1.43)	<0.001
N° days BD^b^	15.38 (15.48)	1.09 (1.60)	18.24 (10.81)	33.42 (11.98)	<0.001
average number of drinks per drinking day^b^	4.99 (3.17)	2.06 (1.69)	6.10 (2.18)	7.88 (2.44)	<0.001
Cannabis units^c^	5.52 (18.26)	0 (0)	0.24 (0.61)	22.37 (31.87)	<0.001
**Outcomes**					
Academic adjustment	6.31 (1.07)	6.55 (1.15)	6.19 (1.07)	6.12 (0.88)	0.10
Personal-emotional adjustment	6.29 (1.27)	6.28 (1.33)	6.29 (1.34)	6.31 (1.10)	0.99
Institutional attachment	7.61 (0.94)	7.65 (0.91)	7.50 (0.98)	7.38 (0.91)	0.48
GPA^d^	6.75 (1.35)	7.18 (1.03)	6.69 (1.40)	6.16 (1.50)	0.002

a The first three items of the alcohol Use Disorders Identification Test.

b In the six prior months to evaluation according to the Alcohol Timeline Follow Back (TLFB).

c In the three prior months to evaluation according to the record of cannabis consumption.

d Grade point average.

### 3.2. Differences between groups in long-term academic adjustment and academic achievement

**Figure 1:**
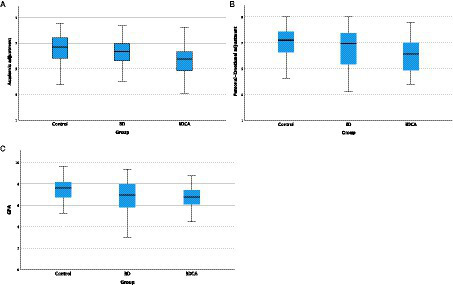
**(A)** Academic adjustment; **(B)** Personal-Emotional adjustment; **(C)** GPA in the different groups.

The ANOVA results (Table 2) show that alcohol and cannabis co-consumption has a negative impact on GPA and is associated with difficulty in adapting to academic demands and personal-emotional problems. Relative to the Control group, the BDCA group showed significantly poorer adjustment to university (*F*_2, 143_ = 7.19, *p* = 0.001), poorer personal-emotional adjustment (*F*_2, 143_ = 4.67, *p* = 0.01) and lower GPAs (*F*_2, 143_ = 3.59, *p* = 0.03) (Figures 1A–C).

**Table 2:** Academic adjustment and GPA in the overall sample and each consumer group.

	Total (*n* = 144)	Control (*n* = 55)	BD (*n* = 54)	BDCA (*n* = 35)	
	*M* (SD)	*M* (SD)	*M* (SD)	*M* (SD)	*p*-value
Academic adjustment	6.19 (1.17)	6.45 (1.27)	6.31 (1.05)	5.57 (0.97)	0.001
Personal-emotional adjustment	6.55 (1.39)	6.88 (1.32)	6.56 (1.46)	5.99 (1.23)	0.011
Social adjustment	6.94 (0.93)	6.73 (1.08)	7.14 (0.77)	6.95 (0.88)	0.068
Institutional attachment	7.73 (0.82)	7.63 (0.88)	7.83 (0.66)	7.73 (0.95)	0.444
GPA	6.95 (1.46)	7.36 (1.37)	6.70 (1.65)	6.71 (1.14)	0.030

Means (M), standard deviations (SD) and associated *p*-value.

BD, Binge Drinking; BDCA, Binge Drinking and Cannabis co-consumption.

### 3.3. Mediation model

**Figure 2:**
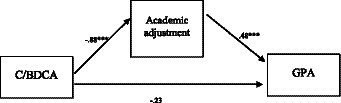
Mediation model with academic adjustment as a mediator between alcohol/cannabis use and GPA (*p < 0.05, **p < 0.01, ***p < 0.001).

The mediation model (Figure 2) shows that the patterns of consumption that students acquire during their first year (T1) have negative effects on their academic performance evaluated at T2. It also showed that academic adjustment explains the mechanism or process whereby the BDCA group performed less well than the C group, mediating the aforementioned relationship. The unstandardized regression coefficients indicate that students with intensive alcohol and cannabis consumption displayed poor academic adjustment (a = −0.88) and that those with poorer academic adjustment performed less well academically (b = 0.48). The indirect effect was statistically different from zero (ab = −0.42, 95% CI = −0.74 to −0.16) representing 64.61% of the total effect (c = −0.65, *p* = 0.021). Furthermore, the results of the mediation model assume the absence of predictor x mediator interactions (*F*_1, 86_ = 0.92, *p* = 0.340).

This significant indirect effect may reflect a causal sequence of events in which a higher consumption (T1) leads to poor academic adjustment and GPA in T1, and therefore the baselines outcomes were included as two covariates in the model. After controlling for academic adjustment and GPA (T1), the indirect effect of consumption on GPA through academic adaptation remained negative and statistically different from zero (effect = −0.17; 95% CI = −0.36, −0.02).

The results of mediation analysis were consistent with the claim that T1 outcome variables were associated with T2 variables. Coefficients shown in Table 3 inform that academic adjustment at T1 was significantly associated with that measured at T2 (0.542, *p* < 0.001) and that GPA at T1 was significantly associated with GPA at T2 (0.536, *p* < 0.001).

**Table 3:** Model coefficient for the mediation analysis with two covariates.

	M (T2 academic adjustment)			Y (T2 GPA)		
	*b*	SE	*p*	*b*	SE	*p*
X (consumption)	−0.606	0.237	0.012	0.212	0.226	0.351
M (T2 academic adjustment)	–	–	–	0.277	0.009	0.006
C1 (T1 academic adjustment)	0.542	0.109	<0.001	0.172	0.114	0.135
C2 (T1 GPA)	0.042	0.094	0.656	0.536	0.086	<0.001
Constant	2.604	0.802	0.002	0.606	0.781	0.440
	*R*^2^ = 0.35			*R*^2^ = 0.52		
	*F*(3, 86) = 15.45, *p* < 0.001			*F*(4, 85) = 23.18, *p* < 0.001		

## 4. Discussion

The time spent at university is a unique opportunity for the intellectual and social growth of student, but it is also a time of high-risk due to exposure to multiple addictive substances with important repercussions for educational aspects and public health (Caldeira et al., 2008; Schulenberg et al., 2017). Existing research on this topic has relied heavily on cross-sectional designs and has mainly focused on the effects of the use of a single psychoactive substance in first-year undergraduates (Aertgeerts and Buntinx, 2002; Liguori and Lonbaken, 2015; Piazza-Gardner et al., 2016). Few studies have used longitudinal designs focused on the simultaneous use of alcohol and cannabis in the university population. This prospective follow-up study helps to improve our knowledge of how consumption of substances may affect academic adjustment and success in Spanish university students. The study findings confirmed the hypothesized association between BD and cannabis co-consumption and poorer academic performance. Our results revealed that the first-year students included in the BDCA group exhibited poorer academic performance in the third year. The interdependent relationship between alcohol and cannabis co-consumption and academic performance documented herein confirms previous findings in cross-sectional (El Ansari et al., 2013; Tembo et al., 2017; Hernández-Serrano et al., 2018; Parisi et al., 2019) and longitudinal (Arria et al., 2015; Suerken et al., 2016; Meda et al., 2017) studies. Thus, the research findings clearly show that poor academic performance is associated with BD and cannabis consumption, whether combined or separate. In one of the few longitudinal studies of this relationship, Meda et al. (2017) found that students who use both substances at moderate to high levels obtained significantly lower grades both at baseline and across the 2 years of research. These results are consistent with those of Arria et al.’s (2015) and Suerken et al.’s (2016), who reported that frequent marijuana use during the first year at college is associated with an increased risk of not completing college or of delayed graduation. The incidence of BD and cannabis use during young adulthood has been associated with to long-lasting neurocognitive alterations (Jackson et al., 2020; Lees et al., 2021). Previous studies have demonstrated that BD and cannabis consumers have poor executive functions and display difficulties in demanding working memory (WM) tasks that require self-monitoring of information (López-Caneda et al., 2014; Gil-Hernandez et al., 2017; Meruelo et al., 2017; Yurasek et al., 2017; Carbia et al., 2018; Deniel et al., 2021). Such deficits can lead to lower academic motivation, poorer study skills, skipping class and disrupted attendance (Arria et al., 2013, 2015).

The second hypothesis was partially confirmed. Consistent with baseline findings (Páramo et al., 2020), the results show that combined BD and cannabis consumption adversely affects academic adjustment. Specifically, we found that academic adjustment was significantly lower in the BDCA group than in control group. BDCA students displayed greater difficulties in dealing with educational demands, such as lower motivation to complete academic tasks, lower academic effort, and greater levels of dissatisfaction with the academic environment. This finding is consistent with those of previous studies (Arria et al., 2013, 2015; Subbaraman and Kerr, 2015; Skidmore et al., 2016; Meda et al., 2017; Jackson et al., 2020), which revealed that, as students who sustained co-consumption tended to experience a decline in their motivation to manage the educational demands of the university experience. It is possible that this low motivation to cope with new academic demands and the greater dissatisfaction with the academic environment may explain the poorer quality of academic adjustment compared to that of non-consumer students. Nevertheless, given that the association between co-consumption and variations in academic adjustment over time in the university population has scarcely been explored, further research is needed before robust conclusions can be drawn.

Another important finding of this study is related to the different developmental pattern in the personal- emotional adjustment dimension. In our first study, we found no differences in this dimension between the BDCA group and the control group. However, in the third year the BDCA group showed poorer personal-emotional adjustment than the students in the control group. As this finding shows, the effect of co-consumption not only interferes at the academic level, but also in the way of experiencing general psychological distress in coping with the demanding requirements of university life. Co-consumption of substances may have cumulative effects and different long-term trajectories (Zucker, 2006; Staff et al., 2010; Arria et al., 2013; Meda et al., 2017; Schulenberg et al., 2017). One possible explanation, similarly to that given in previous studies (e.g., Simons et al., 2005; Hall and Degenhardt, 2009; Keith et al., 2015; Skidmore et al., 2016; Pascoe et al., 2020; De Faria et al., 2021) is that drug use in university students is related to higher levels of avoidance coping, which is related, in turn, to poor sleep quality, poorer well-being, higher perceived stress, irritability and increased symptoms of depression. It is important to note that very few prospective longitudinal studies have focused on understanding the association between co-consumption and personal- emotional adjustment in university students.

Finally, in line with our third hypothesis, the present findings extend our prior research of this cohort sample on the mediating role of academic adjustment in the relationship between combined binge drinking/ cannabis consumption and GPA by the end of the first year of college (Páramo et al., 2020). Academic adjustment remains one of the mechanisms driving the effects of co-consumption on achievement. After 2 years of undergraduate study, the ratio of indirect to total effect (64.61%) was higher than that observed in the first year (34.33%). Furthermore, we also confirmed that the mediating effect of academic adjustment was maintained after controlling for academic adjustment and GPA baseline (T1). Despite the difficulties in explaining academic achievement in co-consumers based on academic adjustment, previous longitudinal (e.g., Wintre et al., 2006) and cross-sectional studies (e.g., Abdullah et al., 2009; Rodríguez et al., 2017; Raza et al., 2021) the already mentioned meta-analysis of 237 studies by Credé and Niehorster (2012) identified academic adjustment as a powerful predictor of university outcomes, or as a mediator of the relationship between drinking motives and alcohol consequences (LaBrie et al., 2012).

### 4.1. Limitations and implications

The current research extends previous findings on BDCA-related consequences for academic adjustment and performance in university students and provides new evidence for a decline in personal-emotional adjustment over time. The results also highlight the important role of adaptation as a key dimension for integration in university life in explaining the association between combined BD/ cannabis consumption and academic performance, even in the context of baseline covariates. Nevertheless, some limitations of the present study should be noted. First, as the sample was only drawn from one university, future studies should be extended to include data from other universities with different characteristics to enable generalization of the results. Second, baseline cannabis and alcohol use had both short-term and long-term impacts on academic outcomes in this sample, but the present findings are limited to a follow-up study with two measurement points (waves). To enhance our understanding of the mechanisms underlying the relationship between substance use and academic performance during university, future research should contemplate taking measurements throughout the entire academic course. Finally, as this study uses a single mediator, further research should assess the impact of other mediating variables on academic performance such as self-esteem (e.g., Li et al., 2018; Tindle et al., 2022) and perceived social support (e.g., Tinajero et al., 2019).

The challenge for future research will be to develop more studies that enable follow-up of changes overtime, enable the appropriate orientation and timing of preventing interventions aimed at combating the problems arising from drug use by students during their time at university. It is therefore necessary to raise awareness in health professionals, educational psychologists, and academic institutions in regarding to the need for support and involvement in prevention, for the provision of guidelines for carrying out suitable intervention strategies.

## 5. Conclusion

The findings of this prospective follow-up study help to advance our knowledge of how co-consumption of substances may affect academic adjustment and achievement in Spanish university students. The results revealed that first year students included in the BDCA group subsequently showed poorer academic adjustment and performance in the third year at university. The effect of co-consumption interferes not only at the academic level but can also lead to general psychological distress in coping with the demanding requirements of university life. We found that in the third year, the BDCA group showed poorer personal-emotional adjustment than the students in the control group. Finally, academic adjustment proved to be one of the mechanisms driving the effects of co-consumption on academic achievement. Overall, the results of this study should encourage health professionals, educational psychologists, and academic institutions to take ownership of the need for support and involvement in prevention, and for provision of guidelines for implementing appropriate intervention strategies.

## Data availability statement

The raw data supporting the conclusions of this article will be made available by the authors, without undue reservation.

## Ethics statement

The studies involving humans were approved by Comité Bioético de la Universidad de Santiago de Compostela. The studies were conducted in accordance with the local legislation and institutional requirements. The ethics committee/institutional review board waived the requirement of written informed consent for participation from the participants or the participants’ legal guardians/next of kin because all participants were of legal age.

## Author contributions

MP and MR: conceived and designed study. MP: writing—original draft preparation. MR: designed and analyzed the data. FC: revised the manuscript and funding acquisition. All authors contributed to the article and approved the submitted version.

## Funding

This work was supported by grants from the Spanish Ministerio de Sanidad, Servicios Sociales e Igualdad, Plan Nacional sobre Drogas (PNSD 2015/034), Ministerio de Economía y Competitividad (PSI2015-70525-P) co-funded for European Regional Development Fund, Ministerio de Ciencia e Innovación (PID2020-113487RB-100) and Xunta de Galicia (GRC ED431C 2017/06; ED431C 2021/08).

## Conflict of interest

The authors declare that the research was conducted in the absence of any commercial or financial relationships that could be construed as a potential conflict of interest.

## Publisher’s note

All claims expressed in this article are solely those of the authors and do not necessarily represent those of their affiliated organizations, or those of the publisher, the editors and the reviewers. Any product that may be evaluated in this article, or claim that may be made by its manufacturer, is not guaranteed or endorsed by the publisher.

## References

[ref1] Abdullah M. C. Elias H. Mahyuddin R. Uli J. (2009). Adjustment amongst first year students in a Malaysian university. Eur. J. Soc. Sci. 8, 496–505.

[ref2] Aertgeerts B. Buntinx F. (2002). The relation between alcohol abuse or dependence and academic performance in first-year college students. J. Adolesc. Health 31, 223–225. doi: 10.1016/s1054-139x(02)00362-2 12225733

[ref3] Allen H. K. Caldeira K. M. Bugbee B. A. Vincent K. B. O’Grady K. E. Arria A. M. (2017). Drug involvement during and after college: estimates of opportunity and use given opportunity. Drug Alcohol Depend. 174, 150–157. doi: 10.1016/j.drugalcdep.2017.01.025, PMID: 28329719 PMC5400721

[ref4] Arnett J. J. (2006). “Emerging adulthood: understanding the new way of coming of age” in Emerging adults in America: Coming of age in the 21st century. eds. Arnett J. J. Tanner J. L. (Washington, DC: American Psychological Association), 85–116.

[ref5] Arnett J. J. Jensen A. J. (2000). Emerging adulthood: a theory of development from the late teens through the twenties. Am. Psychol. 55, 469–480. doi: 10.1037/0003-066X.55.5.469, PMID: 10842426

[ref6] Arnett J. J. Kloep M. Hendry L. Tanner J. (2011). Debating emerging adulthood, stage or process? New York: Oxford University Press.

[ref7] Arria A. M. Allen H. K. Caldeira K. M. Vincent K. B. O’Grady K. E. (2020). Excessive drinking and drug use during college: prospective associations with graduate school plans and attendance. J. Am. Coll. Heal. 68, 132–138. doi: 10.1080/07448481.2018.1535494, PMID: 30763149 PMC6694003

[ref8] Arria A. M. Caldeira K. M. Bugbee B. A. Vincent K. B. O'Grady K. E. (2015). The academic consequences of marijuana use during college. Psychol. Addict. Behav. 29, 564–575. doi: 10.1037/adb0000108, PMID: 26237288 PMC4586361

[ref9] Arria A. M. Garnier-Dykstra L. M. Caldeira K. M. Vincent K. B. Winick E. R. O’Grady K. E. (2013). Drug use patterns and continuous enrollment in college: results from a longitudinal study. J. Stud. Alcohol Drugs 74, 71–83. doi: 10.15288/jsad.2013.74.71, PMID: 23200152 PMC3517265

[ref10] Aspelmeier J. E. Love M. M. McGill L. A. Elliott A. N. Pierce T. W. (2012). Self-esteem, locus of control, college adjustment, and GPA among first- and continuing-generation students: a moderator model of generational status. Res. High. Educ. 53, 755–781. doi: 10.1007/s11162-011-9252-1

[ref11] Bailey T. H. Phillips L. J. (2016). The influence of motivation and adaptation on students’ subjective well-being, meaning in life and academic performance. High. Educ. Res. Dev. 35, 201–216. doi: 10.1080/07294360.2015.1087474

[ref12] Baker R. W. Siryk B. (1989). The student adaptation to college questionnaire. Manual. Western Psychological Services: Los Angeles, CA.

[ref13] Bolin R. M. Pate M. McClintock J. (2017). The impact of alcohol and marijuana use on academic achievement among college students. Soc. Sci. J. 54, 430–437. doi: 10.1016/j.soscij.2017.08.003

[ref14] Busto Miramontes A. Moure-Rodríguez L. Díaz-Geada A. Corral M. Cadaveira F. Caamaño-Isorna F.. (2019). Heavy drinking and non-medical use of prescription drugs among university students: a 9-year follow-up. Int. J. Environ. Res. Public Health 16:2939. doi: 10.3390/ijerph16162939, PMID: 31426271 PMC6720280

[ref15] Caldeira K. M. Arria A. M. O'Grady K. E. Vincent K. B. Wish E. D. (2008). The occurrence of cannabis use disorders and other cannabis-related problems among first-year college students. Addict. Behav. 33, 397–411. doi: 10.1016/j.addbeh.2007.10.001, PMID: 18031940 PMC2247439

[ref16] Carbia C. López-Caneda E. Corral M. Cadaveira F. (2018). A systematic review of neuropsychological studies involving young binge drinkers. Neurosci. Biobehav. Rev. 90, 332–349. doi: 10.1016/j.neubiorev.2018.04.013, PMID: 29678643

[ref17] Clinciu A. I. (2013). Adaptation and stress for the first year university students. Procedia. Soc. Behav. Sci. 78, 718–722. doi: 10.1016/j.sbspro.2013.04.382

[ref18] Coertjens L. Brahm T. Trautwein C. Lindblom-Ylänne S. (2017). Students´ transition into higher education from an international perspective. High. Educ. 73, 357–369. doi: 10.1007/s10734-016-0092-y

[ref19] Crean R. D. Crane N. A. Mason B. J. (2011). An evidence based review of acute and long-term effects of Cannabis use on executive cognitive functions. J. Addict. Med. 5, 1–8. doi: 10.1097/ADM.0b013e31820c23fa, PMID: 21321675 PMC3037578

[ref20] Credé M. Niehorster S. (2012). Adjustment to college as measured by the student adaptation to college questionnaire: a quantitative review of its structure and relationships with correlates and consequences. Educ. Psychol. Rev. 24, 133–165. doi: 10.1007/s10648-011-9184-5

[ref21] Darlington R. B. Hayes A. F. (2017). Regression analysis and linear models: Concepts, applications, and implementation. New York: The Guildford Press.

[ref22] De Faria L. Mezey L. Winkler A. (2021). Cannabis legalization and college mental health. Curr. Psychiatry Rep. 23:17. doi: 10.1007/s11920-021-01231-1 33660096

[ref23] Deniel S. Mauduy M. Cheam-Bernière C. Montcharmont C. Cabé N. Bazire A.. (2021). Why should we ask binge drinkers if they smoke cannabis? Additive effect of alcohol and cannabis use on college students' neuropsychological performance. Addict. Behav. Rep. 14:100362. doi: 10.1016/j.abrep.2021.100362, PMID: 34159250 PMC8202342

[ref24] Derefinko K. J. Charnigo R. J. Peters J. R. Adams Z. W. Milich R. Lynam D. R. (2016). Substance use trajectories from early adolescence through the transition to college. J. Stud. Alcohol Drugs 77, 924–935. doi: 10.15288/jsad.2016.77.924, PMID: 27797694 PMC5088174

[ref25] El Ansari W. Stock C. Mills C. (2013). Is alcohol consumption associated with poor academic achievement in university students? Int. J. Prev. Med. 4, 1175–1188. 24319558 PMC3843305

[ref26] Eskin M. Sun J. M. Abuidhail J. Kujan O. Janghorbani M. Flood C.. (2016). Suicidal behavior and psychological distress in university students: a 12-nation study. Arch. Suicide Res. 20, 369–388. doi: 10.1080/13811118.2015.1054055, PMID: 26954847

[ref27] Fierro Arias D. Moreno Hernández A. (2007). Emerging adulthood in Mexican and Spanish youth: theories and realities. J. Adolesc. Res. 22, 476–503. doi: 10.1177/0743558407305774

[ref28] Gil-Hernandez S. Mateos P. Porras C. Garcia-Gomez R. Navarro E. Garcia-Moreno L. M. (2017). Alcohol binge drinking and executive functioning during adolescent brain development. Front. Psychol. 8:1638. doi: 10.3389/fpsyg.2017.01638, PMID: 29046650 PMC5632721

[ref29] Gómez P. Moure-Rodríguez L. López-Caneda E. Rial A. Cadaveira F. Caamaño-Isorna F. (2017). Patterns of alcohol consumption in Spanish university alumni: nine years of follow-up. Front. Psychol. 8:756. doi: 10.3389/fpsyg.2017.00756, PMID: 28555119 PMC5430027

[ref30] Hall W. Degenhardt L. (2009). Adverse health effects of non-medical cannabis use. Lancet 374, 1383–1391. doi: 10.1016/S0140-6736(09)61037-0 19837255

[ref31] Harvey L. Drew S. Smith M. (2006). The first-year experience: a review of literature for the Higher Education Academy. The Higher Education Academy: York.

[ref32] Hayaki J. Anderson B. J. Stein M. D. (2016). Dual cannabis and alcohol use disorders in young adults: problems magnified. Subst. Abus. 37, 579–583. doi: 10.1080/08897077.2016.1176613, PMID: 27070725 PMC5552039

[ref33] Hayes A. F. (2017). Introduction to mediation, moderation, and conditional process analysis: A regression-based approach, 2nd Edn. New York: The Guilford Press.

[ref34] Hernández-Serrano O. Gras M. E. Font-Mayolas S. (2018). Concurrent and simultaneous use of Cannabis and tobacco and its relationship with academic achievement amongst university students. Behav. Sci. 8:31. doi: 10.3390/bs8030031, PMID: 29494479 PMC5867484

[ref35] Holloway K. Bennett T. H. (2018). Characteristics and correlates of drug use and misuse among university students in Wales: a survey of seven universities. Addict. Res. Theory 26, 11–19. doi: 10.1080/16066359.2017.1309031

[ref36] Jackson K. M. Sokolovsky A. W. Gunn R. L. White H. R. (2020). Consequences of alcohol and marijuana use among college students: prevalence rates and attributions to substance-specific versus simultaneous use. Psychol. Addict. Behav. 34, 370–381. doi: 10.1037/adb0000545, PMID: 31944787 PMC7064425

[ref37] Jacobus J. Squeglia L. M. Infante M. Castro N. Brumback T. Meruelo A. D.. (2015). Neuropsychological performance in adolescent marijuana users with co-occurring alcohol use: a three-year longitudinal study. Neuropsychology 29, 829–843. doi: 10.1037/neu0000203, PMID: 25938918 PMC4633396

[ref38] Jalaliyoon N. Taherdoost H. (2012). Performance evaluation of higher education; a necessity. Proc. Soc. Behav. Sci. 46, 5682–5686. doi: 10.1016/j.sbspro.2012.06.497

[ref39] Johnson R. M. LaValley M. Schneider K. E. Musci R. J. Pettoruto K. Rothman E. F. (2017). Marijuana use and physical dating violence among adolescents and emerging adults: a systematic review and meta-analysis. Drug Alcohol Depend. 174, 47–57. doi: 10.1016/j.drugalcdep.2017.01.012, PMID: 28314193 PMC5521998

[ref40] Johnston L. D. O’Malley P. M. Bachman J. G. Schulenberg J. E. (2009). “Monitoring the future. National survey results on drug use, 1975–2008” in Volume II: College students and adults ages 19–50 NIH publication No. 09-7403 (Bethesda, MD: National Institute on Drug Abuse).

[ref41] Keith D. R. Hart C. C. McNeil M. P. Silver R. Goodwin R. D. (2015). Frequent marijuana use, binge drinking and mental health problems among undergraduates. Am. J. Addict. 24, 499–506. doi: 10.1111/ajad.12201, PMID: 25930151 PMC4551615

[ref42] LaBrie J. W. Ehret P. J. Hummer J. F. Prenovost K. (2012). Poor adjustment to college life mediates the relationship between drinking motives and alcohol consequences: a look at college adjustment, drinking motives, and drinking outcomes. Addict. Behav. 37, 379–386. doi: 10.1016/j.addbeh.2011.11.018, PMID: 22177614 PMC4221271

[ref43] Lees B. Debenham J. Squeglia L. M. (2021). Alcohol and Cannabis use and the developing brain. Alcohol Res. 41:11. doi: 10.35946/arcr.v41.1.11 34567915 PMC8452381

[ref44] Li J. Han X. Wang W. Sun G. Cheng Z. (2018). How social support influences university students' academic achievement and emotional exhaustion: the mediating role of self-esteem. Learn. Individ. Differ. 61, 120–126. doi: 10.1016/j.lindif.2017.11.016

[ref45] Liguori G. Lonbaken B. (2015). Alcohol consumption and academic retention in first-year college students. Coll. Stud. J. 49, 69–77.

[ref46] Liu X. Ping S. Gao W. (2019). Changes in undergraduate students’ psychological well-being as they experience university life. Int. J. Environ. Res. Public Health 16:2864. doi: 10.3390/ijerph16162864, PMID: 31405114 PMC6719208

[ref47] López-Caneda E. Rodríguez Holguín S. Cadaveira F. Corral M. Doallo S. (2014). Impact of alcohol use on inhibitory control (and vice versa) during adolescence and young adulthood: a review. Brit. J. Alcohol Alcohol. 49, 173–181. doi: 10.1093/alcalc/agt168, PMID: 24243684

[ref48] Mallett K. A. Turrisi R. Trager B. M. Sell N. Linden-Carmichael A. N. (2019). An examination of consequences among college student drinkers on occasions involving alcohol-only, marijuana-only, or combined alcohol and marijuana use. Psychol. Addict. Behav. 33, 331–336. doi: 10.1037/adb0000458, PMID: 30869919 PMC6521847

[ref49] Mayhew M. J. Rockenbach A. N. Bowman N. A. Seifert T. A. Wolniak G. C. Terenzini P. T. (2005). “How college affects students” in In 21st century evidence that higher education works (San Francisco: Wiley).

[ref50] Meda S. A. Gueorguieva R. V. Pittman B. Aslanzadeh F. Tennen H. Leen S.. (2017). Longitudinal influence of alcohol and marijuana use on academic performance in college students. PLoS One 12:e0172213. doi: 10.1371/journal.pone.0172213, PMID: 28273162 PMC5342177

[ref51] Merrill J. E. Carey K. B. (2016). Drinking over the lifespan focus on college ages. Alcohol Res. 38, 103–114. PMID: 27159817 10.35946/arcr.v38.1.13 PMC4872605

[ref52] Meruelo A. Castro N. Cota C. Tapert S. (2017). Cannabis and alcohol use, and the developing brain. Behav. Brain Res. 325, 44–50. doi: 10.1016/j.bbr.2017.02.025, PMID: 28223098 PMC5406224

[ref53] Morie K. P. Wu J. Potenza M. N. Mayes L. C. Hammond C. J. Crowley M. J.. (2021). Daily cannabis use in adolescents who smoke tobacco is associated with altered late-stage feedback processing: a high-density electrical mapping study. J. Psychiatr. Res. 139, 82–90. doi: 10.1016/j.jpsychires.2021.05.022, PMID: 34052575 PMC8314801

[ref54] O’Hara R. E. Armeli S. Tennen H. (2016). Alcohol and cannabis use among college students: substitutes or complements? Addict. Behav. 58, 1–6. doi: 10.1016/j.addbeh.2016.02.004, PMID: 26894560 PMC4808449

[ref55] Observatorio Español de la Droga y las Adicciones (OEDA) (2019). Encuesta Estatal Sobre Uso de Drogas en Enseñanzas Secundarias (ESTUDES) 1994–2018. Secretaría de Estado de Servicios Sociales, Delegación del Gobierno Para el Plan Nacional sobre Drogas. Available at: https://pnsd.sanidad.gob.es/profesionales/sistemasInformacion/sistemaInformacion/pdf/ESTUDES_2018-19_Informe.pdf (Accessed December 18, 2019).

[ref56] Páramo M. F. Cadaveira F. Tinajero C. Rodríguez M. S. (2020). Binge drinking, Cannabis co-consumption and academic achievement in first year university students in Spain: academic adjustment as a mediator. Int. J. Environ. Res. Public Health 17:542. doi: 10.3390/ijerph17020542, PMID: 31952153 PMC7014040

[ref57] Parisi C. E. Bugbee B. A. Vincent K. B. Soong A. M. Arria A. M. (2019). Risks associated with alcohol and marijuana use among college student athletes: the case for involving athletic personnel in prevention and intervention. J. Issues Intercoll. Athl. 12, 343–364. PMID: 31588410 PMC6777729

[ref58] Pascoe M. C. Hetrick S. E. Parker A. G. (2020). The impact of stress on students in secondary school and higher education. Int. J. Adolesc. Youth 25, 104–112. doi: 10.1080/02673843.2019.1596823

[ref59] Patte K. A. Qian W. Leatherdale S. T. (2017). Marijuana and alcohol use as predictors of academic achievement: a longitudinal analysis among youth in the COMPASS study. J. Sch. Health 87, 310–318. doi: 10.1111/josh.12498, PMID: 28382670

[ref60] Peer J. W. McAuslan P. (2016). Self-doubt during emerging adulthood. Emerg. Adulthood 4, 176–185. doi: 10.1177/2167696815579828

[ref61] Piazza-Gardner A. K. Barry A. E. Merianos A. L. (2016). Assessing drinking and academic performance among a nationally representative sample of college students. J. Drug Issues 46, 347–353. doi: 10.1177/0022042616659757

[ref62] Raza S. A. Qazi W. Yousufi S. Q. (2021). The influence of psychological, motivational, and behavioral factors on university students' achievements: the mediating effect of academic adjustment. JARHE 13, 849–870. doi: 10.1108/JARHE-03-2020-0065

[ref63] Ribeiro Í. J. Pereira R. Freire I. V. de Oliveira B. G. Casotti C. A. Boery E. N. (2018). Stress and quality of life among university students: a systematic literature review. Health Professions Educ. 4, 70–77. doi: 10.1016/j.hpe.2017.03.002

[ref64] Rodríguez González M. S. Tinajero Vacas C. Guisande Couñago M. A. Páramo Fernández M. F. (2012). The student adaptation to college questionnaire (SACQ) for use with Spanish students. Psychol. Rep. 111, 624–640. doi: 10.2466/08.10.20.PR0.111.5.624-640, PMID: 23234105

[ref65] Rodríguez M. S. Tinajero C. Páramo M. F. (2017). Pre-entry characteristics, perceived social support, adjustment and academic achievement in first-year Spanish university students: a path model. J. Psychol. 151, 722–738. doi: 10.1080/00223980.2017.1372351, PMID: 29023212

[ref66] Sarokhani D. Delpisheh A. Veisani Y. Sarokhani M. Manesh R. Sayehmiri K. (2013). Prevalence of depression among university students: a systematic review and meta-analysis study. Depress. Res. Treat. 2013, 1–7. doi: 10.1155/2013/373857, PMID: 24187615 PMC3800630

[ref67] Saunders J. B. Aasland O. G. Babor T. F. de la Fuente J. U. A. N. R. Grant M. (1993). Development of the alcohol use disorders identification test (AUDIT): WHO collaborative project on early detection of persons with harmful alcohol consumption-II. Addiction 88, 791–804. doi: 10.1111/j.1360-0443.1993.tb02093.x, PMID: 8329970

[ref68] Scales P. C. Benson P. L. Oesterle S. Hill K. G. Hawkins J. D. Pashak T. J. (2016). The dimensions of successful young adult development: a conceptual and measurement framework. Appl. Dev. Sci. 20, 150–174. doi: 10.1080/10888691.2015.1082429 30344455 PMC6176765

[ref69] Schulenberg J. E. Johnston L. D. O’Malley P. M. Bachman J. G. Miech R. A. Patrick M. E. (2017). Monitoring the future National Survey Results on drug use, 1975–2016: 2017 vol. II. College students and adults ages 19–55. Ann Arbor, MI, University of Michigan, Institute for Social Research. Available at: https://deepblue.lib.umich.edu/bitstream/handle/2027.42/139710/mtf-vol2_2016.pdf?sequence=2 (accessed March 15, 2023).

[ref70] Schulenberg J. E. Maggs J. L. (2002). A developmental perspective on alcohol use and heavy drinking during adolescence and the transition to young adulthood. J. Stud. Alcohol Suppl. 14, 54–70. doi: 10.15288/jsas.2002.s14.54 12022730

[ref71] Sharp J. Theiler S. A. (2018). Review of psychological distress among university students: pervasiveness, implications and potential points of intervention. Int. J. Adv. Couns. 40, 193–212. doi: 10.1007/s10447-018-9321-7

[ref72] Simons J. S. Gaher R. M. Correia C. J. Hansen C. L. Christopher M. S. (2005). An affective-motivational model of marijuana and alcohol problems among college students. Psychol. Addict. Behav. 19, 326–334. doi: 10.1037/0893-164X.19.3.326, PMID: 16187813

[ref73] Skidmore C. R. Kaufman E. Crowell S. (2016). Substance use among college students. Child Adolesc. Psychiatr. Clin. N. Am. 25, 735–753. doi: 10.1016/j.chc.2016.06.004 27613349

[ref74] Sobell L. C. Sobell M. B. (1992). “Timeline follow-back: A technique for assessing self-reported alcohol consumption” in Measuring alcohol consumption: Psychosocial and biochemical methods, 41–72.

[ref75] Solowij N. Battisti R. (2008). The chronic effects of cannabis on memory in humans: a review. Curr. Drug Abuse Rev. 1, 81–98. doi: 10.2174/1874473710801010081 19630708

[ref76] Staff J. Schulenberg J. E. Maslowsky J. Bachman J. G. O'Malley P. M. Maggs J. L.. (2010). Substance use changes and social role transitions: proximal developmental effects on ongoing trajectories from late adolescence through early adulthood. Dev. Psychopathol. 22, 917–932. doi: 10.1017/S0954579410000544, PMID: 20883590 PMC2951309

[ref77] Subbaraman M. S. Kerr W. C. (2015). Simultaneous versus concurrent use of alcohol and Cannabis in the National Alcohol Survey. Alcohol. Clin. Exp. Res. 39, 872–879. doi: 10.1111/acer.12698, PMID: 25872596 PMC4399000

[ref78] Substance Abuse and Mental Health Services Administration (2020). Key substance use and mental health indicators in the United States: Results from the 2019 National Survey on drug use and health. Available at: https://digitalcommons.fiu.edu/srhreports/health/health/32/ (Accessed April 15, 2023). 39437027

[ref79] Suerken C. K. Reboussin B. A. Egan K. L. Wagoner K. G. Spangler J. Wolfson M.. (2016). Marijuana use trajectories and academic outcomes among college students. Drug Alcohol Depend. 162, 137–145. doi: 10.1016/j.drugalcdep.2016.02.041, PMID: 27020322 PMC4835174

[ref80] Tembo C. Burns S. Kalembo F. (2017). The association between levels of alcohol consumption and mental health problems and academic performance among young university students. PLoS One 12:e0178142. doi: 10.1371/journal.pone.0178142, PMID: 28658300 PMC5489147

[ref81] Thompson M. Pawson C. Evans B. (2021). Navigating entry into higher education: the transition to independent learning and living. J. Furth. High. Educ. 45, 1398–1410. doi: 10.1080/0309877X.2021.1933400

[ref82] Tinajero C. Cadaveira F. Rodríguez M. S. Páramo M. F. (2019). Perceived social support from significant others among binge drinking and polyconsuming Spanish university students. Int. J. Environ. Res. Public Health 16:4506. doi: 10.3390/ijerph16224506, PMID: 31731610 PMC6888129

[ref83] Tindle R. Abo Hamza E. G. Helal A. A. Ayoub A. E. A. Moustafa A. A. (2022). A scoping review of the psychosocial correlates of academic performance. Rev. Educ. 10:e3371. doi: 10.1002/rev3.3371

[ref84] Varela J. Braña T. Real E. Rial A. (2005). Validación Empírica do AUDIT (Cuestionario de Identificación dos Trastornos debidos ó consumo de alcohol) na Poboación Xeral Galega (Validation of AUDIT for Galician population). Santiago de Compostela: Xunta de Galicia. Consellería de Sanidade-Sergas, 112.

[ref85] Vitale S. A. (2014). “Dope” dilemmas in a budding future industry: an examination of the current status of marijuana legalization in the United States. U. Miami Bus. L. Rev. 23:131

[ref86] Wechsler H. Lee J. E. Kuo M. Seibring M. Nelson T. F. Lee H. (2002). Trends in college binge drinking during a period of increased prevention efforts. Findings from 4 Harvard School of Public Health College alcohol study surveys: 1993-2001. J. Am. Coll. Heal. 50, 203–217. doi: 10.1080/07448480209595713, PMID: 11990979

[ref87] Welsh J. W. Shentu Y. Sarvey D. B. (2019). Substance use among college students. Focus (Am. Psychiatr. Publ.) 17, 117–127. doi: 10.1176/appi.focus.20180037 31975967 PMC6527004

[ref88] Wintre M. G. Bowers C. Gordner N. Lange L. (2006). Re-evaluating the university attrition statistic. J. Adolesc. Res. 21, 111–132. doi: 10.1177/0743558405285658

[ref89] Wolaver A. M. (2002). Effects of heavy drinking in college on study effort, grade point average, and major choice. Contemp. Econ. Policy 20, 415–428. doi: 10.1093/cep/20.4.415

[ref90] Yurasek A. M. Aston E. R. Metrik J. (2017). Co-use of alcohol and Cannabis: a review. Curr. Addict. Rep. 4, 184–193. doi: 10.1007/s40429-017-0149-8, PMID: 32670740 PMC7363401

[ref91] Zucker R. A. (2006). “Alcohol use and the alcohol use disorders: a developmental biopsychosocial systems formulation covering the life course” in Developmental psychopathology: Risk, disorder, and adaptation. eds. Cicchetti D. Cohen D. J. (Hoboken, NJ: Wiley), 620–656.

